# The recovery of upper limb function and postoperative pain in children with lateral humeral condyle fractures were examined retrospectively in relation to the effects of brachial plexus block given in conjunction with general anesthesia

**DOI:** 10.1186/s13018-023-03540-y

**Published:** 2023-03-09

**Authors:** Fan Li, Qiao Yang, Jinrong Yi, Aiqiong Chen

**Affiliations:** Department of Anesthesiology, Ganzhou Maternal and Child Health Hospital, No. 106, Da Road, Zhanggong District, Ganzhou City, 341000 Jiangxi Province China

**Keywords:** Brachial plexus block, General anesthesia, Lateral humeral condyle fracture, Postoperative pain, Upper limb function recovery

## Abstract

**Objective:**

To assess in retrospect the effects of brachial plexus block and general anesthesia on children with lateral humeral condyle fractures in terms of postoperative pain and return of upper limb function.

**Methods:**

Randomly allocated to either the control group (n = 51) or the study group (n = 55) were children with lateral humeral condyle fractures who were admitted to our hospital between October 2020 and October 2021, depending on the surgical anesthetic technique used. The research group had internal fixation surgery with brachial plexus block in addition to anesthesia on the basis of the control group, whereas both groups of children underwent the procedure with general anesthesia alone. Postoperative pain degree, upper extremity functional recovery, occurrence of adverse reactions, etc.

**Results:**

The study group had shorter mean times for surgery, anesthesia, propofol dose, return to consciousness, and extubation than the control group did at every measure of statistical significance. The T2 heart rate (HR) and mean arterial pressure (MAP) were both significantly lower than the pre-anesthesia HR and MAP, and the T1, T2, and T3 HR and MAP were all significantly lower in the study group compared to the control group (*P* < 0.05). The difference between the SpO2 values at T0 and T3 was not statistically significant (*P* > 0.05); the VAS scores at 4 h, 12 h, and 48 h after surgery were higher than those at 2 h after surgery, and reached the peak at 4 h after surgery; within 2 h, 4 h, and 12 h of surgery At 48 h, the study group had substantially lower VAS ratings than the control group (*P* < 0.05). Post-treatment Fugl–Meyer scale scores were considerably higher across the board compared to pre-treatment levels in both groups. When compared to the control group, individuals who participated in the flexion-stretching coordinated exercise and the separation exercise had significantly better ratings. Electrocardiogram, blood pressure, respiratory circulation, and hemodynamic parameters all remained within normal limits during the surgical procedure. The study group had a 9.09% reduced incidence of adverse events compared to the control group. 19.61% (*P* < 0.05).

**Conclusion:**

When used in conjunction with general anesthesia, brachial plexus block can help children with lateral humeral condyle fractures regulate perioperative signs, maintain their hemodynamic level, lessen postoperative pain and unpleasant reactions, and improve the function of their upper limbs. Functional recovery, with high safety and effectiveness.

## Background

A lateral condyle fracture of the humerus is a common type of elbow injury. Because children under 10 years old have a large number of epiphysis at this location, and the lateral malleolus of humerus is used as the cartilage site, which destroys the overall robustness, it is prone to fracture, and the incidence rate of this disease is second to that of supracondylar fracture of humerus, accounting for 16.9% of patients with distal humerus fracture [1, 2]. The main cause of the disease is indirect violence. When falling or severe impact occurs, the elbow joint is in the unbend position or slightly out of the stand, and excessive pressure causes severe traction of the lateral malleolus of humerus, resulting in displacement of the fracture block, and thus, fracture of the lateral malleolus of humerus. If not treated in time, the fracture will not heal, and the nerve will be paralyzed, etc. [3]. Different diagnosis and treatment methods will be adopted for different fracture degrees. The patients without or with mild displacement are usually treated with external fixation, while those with significant displacement are mainly treated with internal fixation by surgery, which can achieve better therapeutic effects [4]. General anesthesia is a common anesthesia method for surgery. Anesthesia drugs are delivered to the respiratory system mainly through surgical intubation, which can stabilize the body hemodynamics, play a role of sedation and analgesia, and improve the compliance of surgery [5]. However, relevant studies [6] have shown that young children with lateral condylar fracture of humerus are difficult to complete anesthesia during operation due to their age, which easily causes unnecessary stress reaction, etc. On the other hand, the anatomical structure of the upper respiratory system of the child is complex, and general anesthesia with airway intubation will damage her laryngeal structure and increase her tension and fear, as well as cause postoperative complications and prolong the postoperative recovery time. Therefore, safe and efficient anesthesia can help the operation smoothly, improve patients' compliance, and then improve the success rate of the operation. Brachial plexus block, as a common anesthesia method in recent years, uses anesthetics to cause nerve conduction block through the nerves in the peripheral nerve trunk of brachial plexus, which has been applied to operations such as upper arm and shoulder, with good anesthesia effect [7]. For the treatment of lateral condylar humerus fractures, the discussion has shifted from the use of brachial plexus block to the selection of a safe general anesthetic, both in China and internationally [8, 9]. Children with a lateral malleolar fracture of the humerus were retrospectively studied to determine the impact of brachial plexus block combined with general anesthesia on perioperative indicators, hemodynamics, postoperative pain intensity, recovery of upper limb function, and adverse responses. The sample for this study consists of 106 occurrences of lateral condylar humerus fractures in children treated at Ganzhou Maternal and Child Health Hospital between October 2020 and October 2021.

## Methods and material

### General material

Children with lateral condylar humeral fractures who were admitted to Ganzhou Maternal and Child Health Hospital between October 2020 and October 2021 made up a total of 106 instances that were used as study participants. The individuals were split into two groups, one receiving the standard surgical anesthetic (n = 51) and the other receiving the experimental anesthesia (n = 55), with the control group included 33 males and 28 females aged 2–10 (mean = 5.26 ± 1.42 years). Participants ranged in age from 2 to 10 years old (5.30 ± 1.41) with the majority (36) being male and the majority (29) being female. Between the two groups, there was no statistically significant difference in demographics like age and gender (*P* > 0.05). Internal fixation surgery was used to treat each kid in each of the two groups. A brachial plexus block was used in conjunction with general anesthesia on the study group, while the control group just got general anesthesia.

### Criteria of inclusion and exclusion

Inclusion criteria: ① Patients were diagnosed with a lateral condylar humeral fracture according to Human Bone Science [10] criteria; ② A lateral condylar fracture of the humerus is visible on imaging examinations like a CT or MRI scan; ③ Individuals between the ages of 2 and 14 with a single-sided lateral condyle humerus fracture; ④ Using the Gartland classification of fracture displacement [11], the fractures were categorized as types II and III; the American Society of Anesthesiologists (ASA) categorized them as types I and II [12]. ⑤ No infection or inflammation at the fracture site; ⑥ Complete clinical data.

Exclusion criteria: ① patients without severe cardiovascular or hepatic or renal dysfunction; ② patients with immune function, coagulation function and severe inflammatory reaction; ③ Patients with cognitive and nervous system disorders; ④ For anesthesia drug allergy, patients with contraindications to surgery; ⑤ Patients with paresthesia and motor nerve injury; ⑥ Patients who did not complete the treatment and were transferred midway.

## Research methods

Internal fixation surgery was used on all the kids in both groups. For the sake of comparison, basic general anesthesia was used on the control group. The patients were informed to fast and drink no more than 6 h before operation, and received intravenous infusion of 0.01 mg/kg atropine injection (Jiangsu Lianshui Pharmaceutical Co., Ltd., NMPN H32020166) and electrolyte 30 min before operation. After that, the children were anesthetized and induced with propofol (Jiangsu Haici Biology Pharmacy Co., Ltd., Yangzijiang Pharmaceutical Group, NMPN H20073642) at 2.0–4.0 mg/kg, sufentanil (Yichang Renfu Pharmacy Co., Ltd., NMPN H20050580) at 0.1–0.2 μ g/kg and vecuronium (Zhejiang Xianju Pharmaceutical Co., Ltd., NMPN H19991172) at 0.08 mg/kg. Sufentanil 8–15 mg/(kg*h) and propofol 5–8 mg/(kg*h) were continuously infused after anesthesia to maintain the BIS at 40–60, and these doses were modified in accordance with the degree of anesthesia. The medication was not given 5 min before surgery. After the consciousness was restored, the airway tube was removed and an appropriate amount of analgesic drugs were given postoperatively.

Based on the experiences of the control group, patients in the study group were given brachial plexus block treatment with anesthesia. The children were instructed to take the supine position before operation, and the fracture site and brachial plexus slave position were detected using Doppler ultrasound instrument. The needle was inserted at 0.5 cm around the target nerve positioned by the probe under ultrasound guidance, and the angle, depth and direction of insertion were adjusted under the guidance of ultrasound images. When there was no blood return by back pumping, 0.5 mL/kg 0.375% ropivacaine hydrochloride (Guangdong CR Shunfeng Pharmaceutical Co., Ltd., NMPN H20050325) was injected for nerve block. After the block, the children were given the same dose of anesthetics. The anesthesia maintenance and listening time were consistent with those in the control group.

### Observational index

① *Perioperative indicators*: The durations of the two groups' children's operations, anesthesias, propofol intakes, regaining consciousness, and extubations were examined and compared. ② *Hemodynamics*: The multi-function monitor (MRNDRAY T8) was used to measure the hemodynamic indexes of the two groups before (T0), during (T1), 15 min(T2) and at the end of the operation (T3) of anesthesia induction, including heartrate (HR), oxygen saturation of pulse oximetry (SpO2), and mean arterial pressure (MAP). ③ *Pain severity after surgery*: A 10 cm ruler was used to quantify the pain intensity of the two groups at 2 h, 6 h, 12 h, and 48 h following surgery using the visual analogue scale (VAS) [13]. A 0 point indicated no pain at all; 1–3 points mean mild pain, and completion has no effect; 4–6 points mean moderate pain and tolerable; 7–9 points of severe pain, unbearable, but improved after taking analgesics; 10 points mean severe pain, unbearable, and useless after medication. ④ Motor recovery in the upper limbs, as assessed by the Fugl–Meyer scale [14]. To assess the motor function of the upper limb both before and after therapy, the Fugl–Meyer Motor Assessment (FMA) was utilized, including limb reflex state, flexion–extension synergistic movement and separation movement of joints of shoulder, elbow, wrist and finger in 33 items, with the score ranging from 0 to 2 points and full score of 66 points, and the score was positively correlated with the motor function of upper limb. ⑤ *Comparison of safety*: ECG, blood pressure, respiratory cycle, and hemodynamics during the perioperative period were tracked, and the adverse reactions, such as nausea, vomiting, restlessness, hypotension, respiratory depression, and drowsiness during this time and in the hospital, were observed and contrasted.

### Statistical analysis

The analysis was conducted using IBM's SPSS 24.0 statistical program. The usual values were measured and represented as $\left( {\overline{x} \pm {\text{s}}} \right)$. T test was used to examine the differences in the data between the two groups. The results of the count were presented in terms of both the raw number of cases and a percentage. The χ2 test was used to do group comparisons.

## Outcome

### Comparison of basic data

Table 1 displays the results, showing that there were no statistically significant differences between the groups regarding gender, age, weight, Gartland type, ASA classification, or the origin of the fractures.

**Table 1:** Comparison of basic data

Group	Control group (n = 51)	Research group (n = 55)	Total value	*P* value
*Gender (cases)*				
Male	28 (54.90)	29 (52.73)	5.060	0.297
Female	23 (54.90)	26 (47.27)		
*Age (years)*				
5.26 ± 1.42	5.30 ± 1.41	4.699	0.384	
Weight (kg)				
16.34 ± 3.51	16.37 ± 3.54	5.187	0.361	
*Gartland type (cases)*				
Type II	27 (52.94)	30 (54.55)	0.254	0.282
Type III	24 (47.06)	25 (45.45)		
*ASA level (cases)*				
Level I	26 (50.98)	28 (50.91)	5.334	0.161
Level II	25 (49.02)	27 (49.09)		
*Fracture causes (cases)*				
Crush	18 (35.29)	20 (36.36)	5.194	0.058
Car accident	21 (41.18)	22 (40.00)		
Others	12 (23.53)	13 (23.64)		

### Comparison of perioperative indicators

The results showed that the operation time, anesthesia time, propofol dosage, consciousness recovery time, Table 2 displays that both the duration of mechanical ventilation and the time it took to extubate patients in the study group were significantly shorter than those of the control group, with a significance level of *P* < 0.05.

**Table 2:** Comparison of perioperative indicators

Group	Control group (n = 51)	Research group (n = 55)
Operation time (min)	157.94 ± 40.26	125.84 ± 34.95^#^
Anesthesia time (min)	113.05 ± 28.68	99.62 ± 23.09^#^
Propofol dosage (mg)	153.72 ± 35.42	110.84 ± 24.15^#^
Consciousness recovery time (min)	14.64 ± 3.10	4.18 ± 1.07^#^
Extubation time (min)	24.19 ± 3.54	6.84 ± 2.12^#^

^#^*P* < 0.05 compared with the Control group

### Comparison of hemodynamic indexes

The study observed no statistically significant changes (*P* > 0.05) in HR, SpO2, and MAP at T0 between the two groups. Patients in both groups experienced increases in HR and MAP at T1, T2, and T3 following anesthesia, in comparison to pre-anesthesia readings, when both were at their lowest. From T1 to T3, the HR and MAP were significantly lower in the study group compared to the control group (*P* < 0.05). Table 3 demonstrates that there was no statistically significant difference in SpO2 between the two groups from time point T0 to time point T3 (*P* > 0.05).

**Table 3:** Comparison of hemodynamic indexes

Group	Time	Control group	Research group
HR (times/min)	T0	72.62 ± 5.02	72.63 ± 5.03
	T1	82.58 ± 7.95*	78.43 ± 6.35*^#^
	T2	79.43 ± 6.39*	75.52 ± 6.12*^#^
	T3	83.74 ± 8.06*	79.33 ± 6.41*^#^
SpO_2_ (%)	T0	98.15 ± 1.15	98.17 ± 1.16
	T1	98.08 ± 1.07	98.11 ± 1.14
	T2	98.34 ± 1.21	98.13 ± 1.15
	T3	98.42 ± 1.28	98.20 ± 1.17
MAP (mmHg)	T0	92.34 ± 5.64	92.37 ± 5.66
	T1	118.91 ± 9.62*	99.76 ± 6.71*^#^
	T2	108.65 ± 7.83*	96.62 ± 6.35*^#^
	T3	111.58 ± 8.37*	98.13 ± 6.53*^#^

*P < 0.05 compared with T0, ^#^*P* < 0.05 compared with the Control group

### Comparison of postoperative VAS scores

After 2 h, there was no statistically significant difference in VAS scores between the two groups (P > 0.05). The highest VAS scores were recorded at 4 h, followed by 12 h, and finally 48 h following surgery, all of which were higher than the VAS values recorded at 2 h. Table 4 shows that after 2 h, 4 h, 12 h, and 48 h after surgery, the VAS ratings of the study group were considerably lower than those of the control group.

**Table 4:** Comparison of postoperative VAS scores

Group	Control group	Research group
After operation 2 h	0.75 ± 0.17	0.70 ± 0.16
After operation 6 h	2.51 ± 0.53*	2.37 ± 0.47*^#^
After operation 12 h	2.32 ± 0.41*	1.96 ± 0.41*^#^
After operation 48 h	1.79 ± 0.26*	1.50 ± 0.09*^#^

*P < 0.05 compared with After operation 2 h, ^#^*P* < 0.05 compared with the Control group

### Comparison of Fugl–Meyer scores

Fugl-scores of Meyer's did not differ significantly (*P* > 0.05) between the two groups before treatment. The scores in the two groups were significantly higher after therapy than they were before treatment for all components of the Fugl–Meyer scale. Research participants improved more than control participants on measures of limb reflex state, joint flexion–extension cooperative movement, and separation movement after the intervention. The results are in Table 5.

**Table 5:** Comparison of Fugl–Meyer scores

Group	Time	Control group	Research group
Limb reflex state	Before treatment	16.62 ± 5.36	16.59 ± 5.34
	After treatment	19.27 ± 5.83*	23.65 ± 6.17*^#^
Joint flexion–extension cooperative movement	Before treatment	17.32 ± 4.67	17.35 ± 4.65
	After treatment	19.36 ± 5.79*	24.07 ± 6.38*^#^
Separation movement	Before treatment	15.17 ± 3.48	15.20 ± 3.51
	After treatment	19.55 ± 4.64*	22.84 ± 6.29*^#^

*P < 0.05 compared with Before treatment, ^#^*P* < 0.05 compared with the Control group

### Comparison of incidence of adverse reactions

During the intraoperative phase, the patient's electrocardiogram (ECG), blood pressure (BP), respiration rate (RR), and hemodynamics all remained within normal ranges. The rate of adverse reactions was 9.09% lower in the study group, at 19.61%, than it was in the control group, at 30.60%. According to Table 6, the difference was highly significant (P < 0.05).

**Table 6:** Comparison of incidence of adverse reactions

Group	Control group	Research group	χ2 value	*P* value
Nausea and vomiting	3 (5.88)	1 (1.82)	–	–
Restlessness	4 (7.84)	2 (3.64)	–	–
Hypotension	1 (1.96)	1 (1.82)	–	–
Respiratory depression	1 (1.96)	1 (1.82)	–	–
Somnolence	1 (1.96)	0 (0.00)	–	–
Incidence of adverse reactions	19.61%	9.09%	5.603	0.036

## Discussion

Immature bone growth, excessive activity, and indirect trauma in children are the most prevalent causes of lateral condylar fractures of the humerus, a common orthopedic illness in children. The main clinical manifestations include swelling, pain and dyskinesia at the lateral elbow joint, as well as tenderness at the lateral condyle of humerus [15]. The early swelling of the fracture site in this disease will cover the fracture symptoms, causing misdiagnosis and missed diagnosis, and then the continuous injury of blood vessels at the fracture end will lead to elbow deformity, bone nonunion, growth disorder, and even bone necrosis, affecting the quality of life in the future [16]. Surgery is the main treatment for lateral condylar fracture of humerus. Both internal and external fixation methods can restore the lateral condylar joint of humerus to the maximum extent and improve the function of elbow joint, with significant therapeutic effect [17]. However, during the operation, it was found that due to incomplete mental development, fear and resistance to the operation as well as behaviors, it was difficult for the children to cooperate with the surgical anesthesia, and the operation could not be performed smoothly, which led to stress damage and the expected prognosis could not be achieved [18]. Therefore, the choice of surgical anesthesia is an important factor to ensure the smooth operation. General anesthesia and brachial plexus block, as the common anesthesia means for orthopedic surgery, can improve the compliance during the operation and obtain good anesthesia effect, but the research on the postoperative pain severity and upper limb functional recovery is still limited [19, 20]. In this paper, brachial plexus block combined with general anesthesia intervention is adopted for children with lateral condylar fracture of humerus. It can improve perioperative indicators, stabilize hemodynamic indicators, relieving postoperative pain and adverse reactions, improving upper limb function and living standard, and achieving significant anesthesia effect.

Internal fixation of humeral lateral condyle fracture is a traumatic operation, which will cause stress reaction and hemodynamic fluctuation, which will have a negative impact on the smooth operation. Besides, after operation, the pain degree will be increased due to surgical stimulation, which is not conducive to the recovery of prognosis. Adopting a feasible anesthesia scheme can promote the smooth operation and maintain the fluctuation range of hemodynamics [21]. Wang et al. [22] treated patients with lower limb fracture with ketamine combined with nerve block intervention, which can significantly stabilize the fluctuation of HR and MAP levels, improve the success rate of nerve block, and have high safety; Town C J et al. [23] used ultrasound-guided brachial plexus block to treat burn patients, which can reduce their VAS scores and relieve their pain. In this study, the study group's surgery, anesthesia, propofol dose, recovery from unconsciousness, and extubation times were shorter than those of the control group. Both groups' HR and MAP levels were higher after anesthesia than they were before it, with the HR and MAP levels in T2 being at their lowest. The study group's HR and MAP levels were also lower than those in the control group's, while the difference between the two groups' SpO2 levels in T0 through T3 was not statistically significant. The VAS ratings peaked at 4 h after surgery, and were greater after 4 h, 12 h, and 48 h than they were at 2 h. The VAS ratings of the study group were significantly lower than those of the control group at 2 h, 4 h, 12 h, and 48 h post-surgery. The findings largely support those of Wang J. and Town C. J., showing that brachial plexus block and general anesthetic intervention may successfully maintain hemodynamic fluctuation during surgery without escalating postoperative discomfort. The reason may be that brachial plexus block can directly contact and block anesthetic drugs with targeted nerves under the condition of ultrasonic detection, reduce the dosage of anesthetic drugs, reduce the stimulation to other organs and tissues, stabilize the hemodynamic level of children during operation, and shorten the onset time of anesthesia and the recovery time after operation. Applying brachial plexus block on the basis of mastering the anatomical structure of the blood vessels, tissues and muscles around the brachial plexus of children can improve the accuracy of puncture during surgery, avoiding repeatedly adjusting the angle and depth of needle insertion, improving the compliance of children during surgery, and the success rate of anesthesia, reducing the stress response of the body as much as possible, and relieving the degree of pain during surgery [24, 25].

Internal fixation during surgery allows the cartilage tissue that makes up the shattered parts of the lateral condyle of the humerus to continue developing, expanding, and calcifying. Children won't be able to participate with the procedure because of their anxiety and worry, which will cause more tissue damage surrounding the fracture area and make it difficult for the procedure to go smoothly, impairing the function of the upper limbs [26]. The study's findings demonstrate that two groups of children's Fugl–Meyer scores are considerably higher after therapy than they were before. Following therapy, the study group's ratings for joint flexion and extension movement, as well as separation movement, are greater than those of the control group. Perioperative ECG, blood pressure, respiratory circulation, and hemodynamics were not noticeably aberrant, and the study group experienced less adverse effects than the control group did. Brachial plexus block combined with general anesthesia intervention can reduce the damage to upper limb function and reduce the risk of adverse reactions. In this study, brachial plexus block combined with general anesthesia is adopted. When the children are awake, anesthesia drugs are given, which can reduce the stress stimulation of the children to the operation, and block the pain degree after the anesthesia drugs take effect. The puncture process is safe and efficient, which is conducive to stabilizing the children's mood and pain, and promoting their cooperation with the operation. General anesthesia allows anesthetic drugs to enter the body through intravenous or airway intubation, which can maintain the vital signs, achieve a painless and unconscious state, improve the anesthesia effect, and do not worry about the children's uncooperative, which is conducive to the recovery of upper limb function after surgery; In addition, ultrasound-guided brachial plexus block can detect the pharmacokinetics of anesthetic drugs in brachial plexus, master its expansion and anesthetic effect, rationally applying anesthetic drugs, avoiding excessive accumulation of anesthetic drugs and incomplete metabolism. It reduces the incidence of nausea and vomiting, respiratory depression, etc., improving postoperative living standards, and promoting the recovery of upper limb function [27, 28].

There are still some limitations in this study, mainly in the limited number of samples, and there may be some deviation between clinical results and research data, which will affect the reliability of research results. With the occurrence of postoperative adverse reactions, the anesthetic pharmacist did not count the differences in the anatomical structure and puncture echo of the surgical site of the children, which would all affect the postoperative pain. This study is a retrospective analysis, and its influence on diagnosis, treatment and prognosis cannot be accurately judged. Therefore, it needs to be further verified in a prospective, multicenter study.

To sum up, the intervention of brachial plexus block combined with general anesthesia for children with lateral condylar fracture of humerus has obvious sedative and analgesic effects, maintaining stable hemodynamics, relieving stress reaction during operation, which is conducive to the recovery of upper limb function. It reduces the incidence of adverse events such as respiratory depression, and lays a foundation for the smooth operation.

## Data Availability

Data are available from the corresponding author on request.
